# The Use of Short Segment Free Jejunal Transfer as Salvage Surgery for Cervical Esophageal and Hypopharyngeal Cancer

**DOI:** 10.1007/s00268-013-2229-9

**Published:** 2013-10-01

**Authors:** Shuhei Mayanagi, Tetsuro Onitsuka, Masahiro Nakagawa, Hiroshi Sato, Yuko Kitagawa, Yasuhiro Tsubosa

**Affiliations:** 1 Division of Esophageal Surgery, Shizuoka Cancer Center Hospital, 1007 Shimonagakubo, Nagaizumi-cho, Sunto-gun, Shizuoka 411-8777 Japan; 2 Department of Surgery, Keio University School of Medicine, Tokyo, Japan; 3 Division of Head and Neck Surgery, Shizuoka Cancer Center Hospital, Shizuoka, Japan; 4 Division of Plastic and Reconstructive Surgery, Shizuoka Cancer Center Hospital, Shizuoka, Japan; 5 Department of Esophagogastric Surgery, Saitama Medical University International Medical Center, Saitama, Japan

## Abstract

**Background:**

Salvage surgery after definitive chemoradiotherapy for cervical esophageal cancer and hypopharyngeal cancer remains a challenge because of the high rate of complications. The purpose of this study was to evaluate the safety and efficacy of free jejunal transfer as salvage surgery for cervical esophageal cancer and hypopharyngeal cancer after definitive chemoradiotherapy.

**Methods:**

We enrolled eight patients with cervical esophageal cancer and 11 patients with hypopharyngeal cancer who underwent free jejunal transfer as salvage surgery following radiotherapy or chemoradiotherapy. In this study, we reviewed the surgical procedures, perioperative complications, and survival rates.

**Results:**

The median duration of surgery was 514 min, and the median blood loss was 439 ml. In surgical procedures, the recipient vessels for the anastomosis of the free jejunum consisted of one artery and one vein (63 %), one artery and two veins (5 %), and two arteries and two veins (31 %). The postoperative morbidity rate was 57.9 % (11 patients), with six cases of partial necrosis of the tracheal margin and no cases of graft necrosis or postoperative in-hospital death. The overall 5-year survival rate after surgery was 58.1 %.

**Conclusions:**

Our findings suggest that with careful attention to the potential development of necrosis of the tracheal margin, pharyngolaryngoesophagectomy and free jejunal transfer can be safely performed, even in patients who received radiotherapy or chemoradiotherapy.

## Introduction

Cervical esophageal cancer is relatively uncommon, accounting for less than 5% of all esophageal cancers [1]. Hypopharyngeal cancer also accounts for approximately 6 % of all head and neck cancers [2]. In recent years, chemoradiotherapy (CRT) has been accepted as the standard initial therapy for cervical esophageal and hypopharyngeal cancers [3]. Salvage surgery has subsequently become important, because it allows for organ preservation. Patients often choose definitive CRT as the initial treatment. When a patient requires additional treatment for recurrent or residual lesions, salvage surgery is the only treatment for a cure or long-term disease control. However, surgery after irradiation is far more challenging compared with primary surgery, because the risk of operative morbidity and mortality increases when there is a history of CRT [4, 5]. Various surgical procedures have been attempted for reconstruction to improve the performance of surgery. Since Seidenberg reported a technique to reconstruct the cervical esophagus by free jejunal transfer (FJT) of a revascularized, isolated jejunal segment [6], it has become one of the standard techniques used for reconstruction after total pharyngolaryngoesophagectomy (TPLE). However, to the best of our knowledge, few studies have reported the efficacy and safety of salvage FJT after CRT [7–9]. We hypothesized that FJT could be a surgical procedure as safe and effective as salvage surgery following CRT. In this study, we report the outcomes of 19 patients who underwent FJT, and we outline the reconstructive strategy we performed at our institute.

## Patients and methods

From September 2002 to July 2012, a total of 108 patients underwent TPLE for cervical esophageal and hypopharyngeal cancers at the Shizuoka Cancer Center Hospital (Shizuoka, Japan). We excluded 89 patients for whom radical surgery was the initial treatment. Thus, we included 19 patients who received definitive CRT or radiotherapy (RT), followed by salvage TPLE and FJT. We defined a dose of definitive RT as more than 50 Gy. Patients were evaluated preoperatively by using endoscopy, ultrasonography, computed tomography, and magnetic resonance imaging. The clinical staging and pathological examination for resected lesions was performed according to the TNM classification. Medical records were analyzed retrospectively to determine clinical characteristics and surgical outcomes, including recipient vessels, number of dissected lymph nodes, operative time, blood loss, duration from CRT to surgery, postoperative hospital days, and complications. This study was approved by the institutional review board of Shizuoka Cancer Center Hospital.

## Pharyngolaryngoesophagectomy and free jejunal transfer

**Fig. 1:**
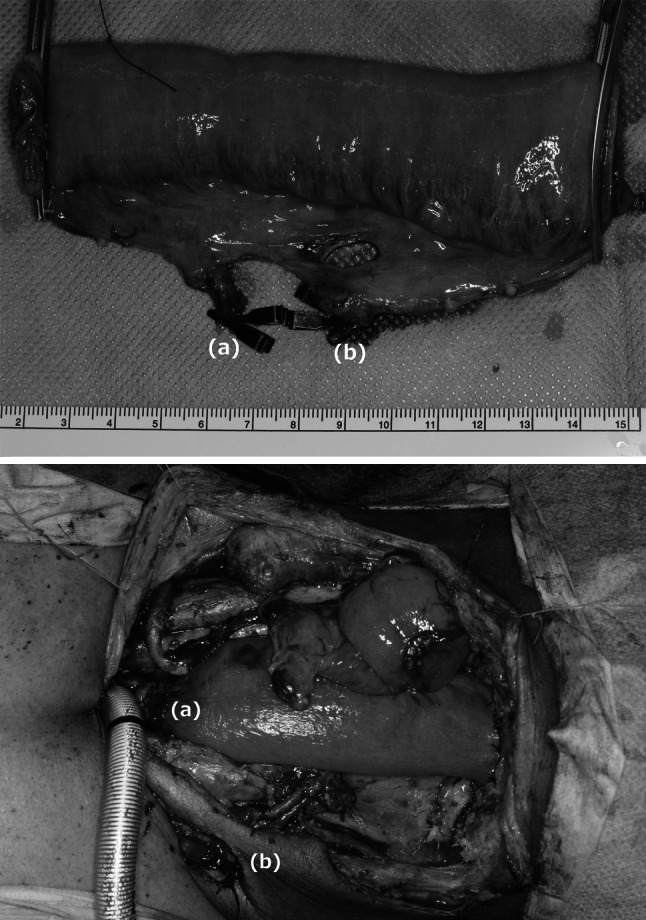
Free jejunal transfer. *Top* The harvested free jejunal graft had two vascular pedicles; a second jejunal artery/vein (*a*) and a third jejunal artery/vein (*b*). *Bottom* The second jejunal artery was anastomosed to the right superficial cervical artery (*a*). The third jejunal artery was anastomosed to the left superficial cervical artery (*b*)

The Y-shaped skin incision was placed so that upper and bilateral lower skin flaps were formed. We dissected a minimal possible area of the enlarged lymph nodes before definitive CRT or RT. The strap muscles were cut off at the insertion of sternum to exteriorize the thyroid after conservative neck dissection. If at all possible, we left the thyroid with anterior and inferior thyroid artery and kept a connection between esophagus and membranous wall of trachea without compromising tracheal vascularity. To maintain the microvascular blood flow, we preserved the tissue surrounding the trachea when we performed TPLE. We used a free jejunum, approximately 15 cm in length, with the second or/and third jejunal vessels (Fig. 1). The jejunal mesenteric vessels were first evaluated by using transillumination. If the harvested jejunum was supplied by a double vascular pedicle, adequate circulation of the free jejunal graft should be confirmed by pulsation of the collateral arteries by the atraumatic clamp test before cutting off the vessels. To reduce the possibility of necrosis in the free jejunum, we selected a single or double vascular anastomosis, depending on clamp test results and shape of the jejunal vessels. Before revascularization, we performed an anastomosis between the free jejunum and esophagus using a vertical mattress suture for the posterior wall and Gambee suture for the anterior wall. We performed an end-to-side anastomosis of the donor vessel to the internal jugular vein and subsequently performed an end-to-end arterial anastomosis. Finally, an anastomosis of the pharynx and jejunum was performed using sutures similar to those used for the jejuno-esophageal anastomosis. A longitudinal incision was made at the corner of the free jejunum corresponding to the midpoint of the front wall of the pharyngeal stump to adjust the difference in diameter between the pharyngeal defect and free jejunal graft. The tracheostoma was made at the inferior end of longitudinal Y-shaped skin incision. The trachea, having been mobilized, was brought up and held against the skin. To prevent a stenosis of the tracheostoma, we employed elevation of triangular skin flap, removal of a triangular section of anterolateral tracheal wall, and sutured flap into the apex of the triangle. We sutured the skin to the trachea without passing through tunica mucosa of trachea by 4-0 monofilament absorbable suture. Enteric nutrition through jejunostomy or a nasogastric feeding tube also was used routinely.

## Results

The clinical characteristics of the patients are shown in Table 1. The patients who underwent TPLE and FJT had a median age of 65 years. One patient received RT alone as the initial treatment, whereas the others were treated with concurrent CRT. All patients who underwent CRT received a platinum-based regimen, except one case that was treated using an unspecified regimen. The median dose of radiation was 66 Gy.

**Table 1:** Patient characteristics

Age (year), median (range)	65	(49–77)
Gender		
Male	17	89 %
Female	2	11 %
Anatomical subsite		
Cervical esophagus	8	42 %
Hypopharynx	11	58 %
Preoperative diagnosis		
Cervical esophagus		
Stage IIA	1	5 %
Stage III	6	32 %
Stage IVB	1	5 %
Hypopharynx		
Stage I	1	5 %
Stage III	2	11 %
Stage IVA	8	42 %
Preoperative treatment		
RT alone	1	5%
CRT		
5-FU+cisplatin+RT	7	37 %
5-FU+carboplatin+RT	2	11 %
5-FU+nedaplatin+RT	1	5 %
Cisplatin+RT	7	37 %
Unspecified regimen + RT	1	5 %
Dose of radiation (Gy), median (range)	66	(58–70)

*RT* radiotherapy, *CRT* chemoradiotherapy, *FU* fluorouracil

**Fig. 2:**
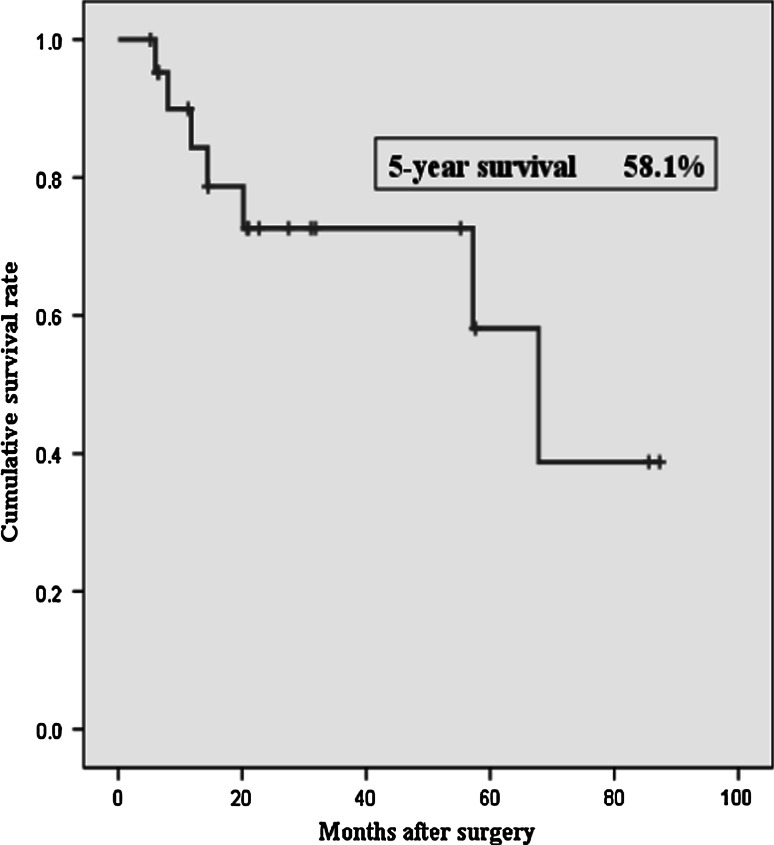
Disease-specific survival curve following salvage surgery

The data regarding surgical outcomes and postoperative complications are shown in Table 2. The recipient vessels consisted of one artery and one vein (63 %), one artery and two veins (5 %), or two arteries and two veins (31 %). The recipient arteries were as follows: the superior thyroid artery (ten patients, 53 %); superficial cervical artery (six patients, 32 %); lingual artery (five patients, 26 %), and transverse cervical artery (three patients, 16%). The median duration of surgery was 514 min; blood loss was 439 ml. The postoperative morbidity rate was 57.9 % (11 patients), with six cases of partial necrosis of the tracheal margin, and no cases of graft necrosis or postoperative in-hospital death. The median time from initial therapy to salvage surgery was 218 days. The rate of local recurrence and distant metastases after salvage surgery were 21 % (four patients) and 42 % (eight patients), respectively. The overall 5-year survival rate after salvage surgery was 58.1 % (Fig. 2).

**Table 2:** Surgical outcomes

Length of operation (min), median (range)	514	(329–739)
Intraoperative blood loss (ml), median (range)	439	(80–1,430)
Duration from CRT to surgery (days), median (range)	218	(88–3,043)
Postoperative hospital (days), median (range)	18	(14–38)
No. of dissected lymph nodes, median (range)	7	(0–38)
Recipient vessels for free jejunal transfer		
One artery and one vein		
STA	7	37 %
SCA	2	11 %
LA	2	11 %
TCA	1	5 %
One artery and two veins		
TCA	1	5 %
Two arteries and two veins		
LA + SCA	2	11 %
LA + STA	1	5 %
STA + SCA	1	5 %
STA + TCA	1	5 %
Bilateral SCA	1	5 %
Complications		
Partial necrosis of tracheal margin	6	32 %
Anastomotic stenosis	3	16 %
Fistula	1	5 %
Abdominal incisional hernia	1	5 %
Wound infection	1	5 %

*RT* radiotherapy, *CRT* chemoradiotherapy, *FU* fluorouracil, *STA* superior thyroid artery, *SCA* superficial cervical artery, *LA* lingual artery, *TCA* transverse cervical artery

## Discussion

In the present study, we reviewed the surgical procedures, postoperative complications, and prognosis of patients who underwent salvage TPLE and FJT for cervical esophageal and hypopharyngeal cancers. Our findings showed that salvage TPLE and FJT could be safely performed, even in patients who have received CRT, with careful attention to the potential development of necrosis of the tracheal margin. Our findings suggested that salvage surgery for cervical esophageal and hypopharyngeal cancers may improve the prognosis for patients, even for those who have residual or recurrent tumors after CRT.

Locoregional recurrences are observed in 15–50 % of the patients with squamous cell carcinoma of the head and neck involving the cervical esophagus, and this is a major factor contributing to death [10, 11]. Survival is poor for most patients with recurrent disease, and the therapeutic options are limited because of previous treatment. The weighted average 5-year survival in a meta-analysis was 39 % in patients who received salvage surgery for head and neck cancer [12]. Factors, such as the stage of recurrence and disease-free interval, have a significant effect on prognosis. Therefore, if the recurrent disease is amenable to resection and the patient is medically operable, salvage surgery provides the best opportunity for long-term survival.

Few studies have reported the outcome of salvage TPLE after definitive CRT for cervical esophageal and hypopharyngeal cancer [7–9, 13]. These studies reported high morbidity and mortality rates associated with salvage surgery. The reported rate of complications ranged from 21 to 43 % with the median and upper limit of radiotherapy ranged from 65.6 to 70 Gy and from 71 to 74 Gy, respectively. In particular, graft necrosis due to thrombosis, kinks, etc., following vascular anastomosis requires secondary reconstructive surgery. Moreover, it often is difficult to use arteries within an irradiated area as recipient vessels [14, 15]. Miyamoto et al. [7] reported a recipient-site complication rate of 20.9 % and perioperative death of 2.3 % in their series of 86 patients with median irradiation dose of 70 (range 38–72) Gy. The major complications of recipient-site were anastomotic thrombosis (4.7 % of cases), fistula (3.5 % of cases), loss of the jejunum (3.5 % of cases), skin necrosis (2.3 % of cases), and carotid rupture (2.3 % of cases). In their study, the median dose and upper limit of radiotherapy were 65.6 and 71.5 Gy, respectively. In this study, the median of radiation was 66 Gy. At least less than 66 Gy, our strategy could be safely performed without a significant increase in morbidity or mortality. In previously irradiated necks, the identification and dissection of a suitable recipient artery was a tedious procedure. It required delicate dissection to avoid unnecessary destruction of potential vessels during tumor excision and lymph node dissection and must be performed by using a surgical microscope during the anastomosis.

A double pedicled jejunum transfer method is safe theoretically and reduces the possibility of losing the graft [16, 17]. The first 60 cm of the jejunum is used as a graft, and has three jejunal arteries on average (range 1–5 jejunal arteries), with 3–5 arteries present in 84 % cases [18]. Free jejunal grafts often have double pair of pedicles, and these mesenteric vessels connect with each other, forming a vascular loop through collateral vessels. Thus, we developed an intraoperatively appropriate revascularization procedure and adopted a double pedicled jejunum transfer in patients with unreliable vessels. The use of recipient arteries from a different flow-sourced carotid system (superior thyroid and lingual artery) and subclavian system (superficial cervical and transverse cervical artery) has the potential to increase blood supply to a graft. However, when a single donor artery/vein pair has an adequate vessel caliber, the results are satisfactory and there is no loss of the graft.

Because irradiation to the neck may damage the tracheal sheath, necrosis of the tracheal margins is a possible complication of TPLE, particularly in patients undergoing preoperative CRT. Protective measures to prevent ischemic tracheal lesions include careful dissection around the airway. Intraoperatively, the aim should be for an R0 resection. To preserve blood supply to the trachea, this should be achieved using a conservative nodal dissection rather than a radical dissection in the irradiated field. Not only irradiation but also irradiation field has an effect on tracheal necrosis. Salass et al. [19] mentioned that blood supply of the trachea was dependent on preservation of the lateral tracheal vascular pedicles that were derived from branches of the inferior thyroid, supreme intercostal, subclavian, internal mammary, innominate, and bronchial arteries. These vessels interconnect longitudinal tracheal anastomosis. Moreover, anastomoses in and around the thyroid gland supplement the circulation to the cervical trachea, with contributions from the superior thyroid artery. Especially for extensively invasive cervical esophageal cancer, there is the potential for a decrease in blood flow to residual trachea due to extended field irradiation to longitudinal arterial anastomosis. If possible, one of four right and left affiliates of the bloodstream from either the superior thyroid artery with thyroid gland or the inferior thyroid artery should be kept to prevent a blood supply to trachea. It also is important to keep a connection between esophagus and membranous wall of trachea. If possible, we left the thyroid with anterior and inferior thyroid artery and kept a connection between esophagus and membranous wall of trachea. We dissected the minimal area of the enlarged lymph nodes before definitive CRT. Our results suggest that a minimally invasive neck dissection may improve the postoperative survival rate without severe complications.

For long period of time, pedicled myocutaneous flaps have been used in the prevention of potential wound breakdown, when compromised healing can be anticipated at reconstruction site of TPLE (Table 3) [7, 8, 20–26]. To cover the jejunal graft, intestinal anastomoses, and exposed great vessels of the neck with nonirradiated well-vascularized tissue, a myogenous, denervated pectoralis major muscle was raised and fixed in the neck. It became evident that a pectoralis major muscle flap might help to diminish complications stemming from the reconstruction followed by TPLE [25, 26]. Recently, most pharyngoesophageal defects were reconstructed with the free jejunum or free myocutaneous flap, the radial forearm flap [23, 24], and anterolateral thigh flap [20]. Yu et al. [20] reported that the anterolateral thigh flap offers many advantages for pharyngoesophageal reconstruction compared with traditional flaps. It resulted in excellent speech quality and swallowing function, minimal donor site morbidity, and quick postoperative recovery. However, one of the frequent criticisms of the myocutaneous flap was the difficulty in working difficulty for construction in obese patients because of too thick flap. In point of swallowing function, the isoperistalsis of the jejunum flap may help swallowing transit compared with the myocutaneous flap reconstruction. Our results suggest that FJT after TPLE should be the initial reconstruction of choice, especially for salvage surgery, because of its low rate of major complications.

**Table 3:** Comparison of reported studies for complications

Author	Patients	Reconstruction type	Previous radiation (%)	Fistula (%)	Stenosis (%)	Flap loss (%)	Mortality rate (%)
Our study	19	FJT	100	5	16	0	0
Miyamoto et al. [7]	86	FJT	100	12	NA	4	2
Kadota et al. [8]	40	FJT	100	13	0	5	0
Yu et al. [20]	114	ATF	67	9	6	2	2
Murray et al. [21]	14	ATF	21	0	14	0	0
Genden et al. [22]	12	ATF	83	9	9	8	0
Azizzadeh et al. [23]	20	RFFF	85	20	20	0	0
Scharpf et al. [24]	28	RFFF	72	28	36	0	0
Genden et al. [22]	11	RFFF	73	8	27	0	0
Zbar et al. [25]	24	PMMF	100	NA	NA	13	0
Dubsky et al. [26]	8	FJT and PMMF	71	0	13	0	0

*FJT* free jejunal transfer, *PMMF* pectoralis major myofascial flap, *RFFF* radial forearm free flap, *ATF* anterolateral thigh flap, *NA* data not quoted or not possible to interpret accurately

Despite the potential for complications, several patients have achieved an improved quality of life following surgery [12]. The use of salvage surgery for patients with an esophageal obstruction or stricture after CRT can relieve dysphagia and restore the ability to swallow. As more patients are treated with definitive CRT for functional organ preservation, there may be an increased role for surgical salvage. Advances in reconstructive surgery, particularly the use of microvascular-free flaps, have allowed more patients to be candidates for salvage surgery.

Salvage TPLE and FJT can offer the exclusive chance of prolonged survival in patients who have locoregional failure after CRT. However, salvage surgery is known to be a highly invasive procedure and is associated with various postoperative complications compared with primary surgery without antecedent radiotherapy. Thus, the option for salvage surgery must be carefully considered to maintain the balance between curability and safety.
